# Risk factors for prehypertension and their interactive effect: a cross- sectional survey in China

**DOI:** 10.1186/s12872-018-0917-y

**Published:** 2018-09-15

**Authors:** Jian Song, Xue Chen, Yingying Zhao, Jing Mi, Xuesen Wu, Huaiquan Gao

**Affiliations:** 1 School of public health, Bengbu medical college, 2600 Donghai Road, Bengbu, 233000 Anhui Province China; 2 Bengbu health board, 568 Nanhu Road, Bengbu, 233000 Anhui Province China

**Keywords:** Prehypertension, Visceral obesity, LAP, Interaction

## Abstract

**Background:**

Individuals with prehypertension are at higher risk of developing hypertension and cardiovascular diseases, while the interaction between factors may aggravate prehypertension risk. Therefore, this study aimed to evaluate the risk factors for prehypertension in Chinese middle-aged and elderly adults, and explore the potentially interactive effect of evaluated factors.

**Methods:**

All the participants that came from a community based cross-sectional survey were investigated in Bengbu, China, by being interviewed with a questionnaire. Body mass index (BMI), Waist circumference (WC) and lipid accumulation product (LAP) that reflect participants’ obesity were also calculated. In addition, logistic regression model was applied to explore the risk factors of prehypertension, followed by the assessment of the interactive effects between risk factors on prehypertension by the relative excess risk due to interaction (RERI), attributable proportion due to interaction (AP) and synergy index (SI).

**Results:**

A total of 1777 participants were enrolled in this study, among which the prevalence of normtension, prehypertension and hypertension were 41.70%, 33.93% and 24.37% respectively. According to the multivariate logistic regression analysis, age (OR: 1.01, 95%CI: 1.00–1.02), smoking (OR: 1.67, 95%CI: 1.22–2.29), family history of cardiovascular diseases (OR: 1.52, 95%CI: 1.14–2.02), general obesity (OR: 1.51, 95%CI: 1.15–1.97) and LAP (OR: 2.58, 95%CI: 1.76–3.80) were all defined as the major factors that significantly related with the risk of prehypertension. When identifying prehypertension risk, the receiver-operating characteristics (ROC) curves (AUC) analysis indicated that LAP performed better than BMI in males (*Z* = 2.05, *P* = 0.03) and females (*Z* = 2.12, *P* = 0.03), but was superior to WC only in females (*Z* = 2.43, *P* = 0.01). What is more, there were significant interactive effects of LAP with family history of cardiovascular diseases (RERI: 1.88, 95%CI: 0.25–3.51; AP: 0.44, 95%CI: 0.20–0.69; SI: 2.37, 95%CI: 1.22–4.60) and smoking (RERI: 1.99, 95%CI: 0.04–3.93; AP: 0.42, 95%CI: 0.17–0.67; SI: 2.16, 95%CI: 1.68–4.00) on prehypertension risk. The value of AP (0.40, 95%CI: 0.03–0.77) also indicated a significant interaction between family history of cardiovascular diseases and smoking on prehypertension.

**Conclusion:**

Prehypertension is currently prevalent in Chinese adults. This study indicated that age, family history of cardiovascular diseases, smoking, general obesity and LAP were significantly related with prehypertension risk. Furthermore, interactive effects on risk of prehypertension had been demonstrated in this study as well, which would help researchers to build strategy against prehypertension more comprehensively and scientifically.

**Electronic supplementary material:**

The online version of this article (10.1186/s12872-018-0917-y) contains supplementary material, which is available to authorized users.

## Background

Elevated blood pressure is a common and serious public issue [1, 2], which was significantly related to incidence of gout [3], Parkinson’s disease [4] and even prognosis in patients with systemic lupus erythematosus [5]. In 2003, the definition of prehypertension was first proposed in the Seventh Report of the Joint National Committee on Prevention, Detection, Evaluation, and Treatment of High Blood Pressure (JNC-7) [6]. According to JNC-7, prehypertension was more likely to develop hypertension than normotension [6]. Soon afterwards, relevant studies have demonstrated that the interventions against prehypertension may bring new breakthroughs in the prevention of hypertension [7, 8]. With the social development and acceleration of the population aging in China, the prevalence of prehypertension and hypertension has significantly increased. As indicated in a large-scale multi- ethnic population survey with 47,495 adult participants in China, the prevalence of prehypertension was up to 36.4% [9]. Moreover, a clustering of cardiovascular disease risk factors was also observed in the prehypertension population of Han and Mongolian adults [10]. A four-year follow-up study indicated that prehypertension significantly increased the occurrence of chronic kidney disease in Chinese adults [11]. Meta-analyses have demonstrated that prehypertension, as well as hypertension and diabetes, are significantly associated with the risk of cardiovascular events including coronary heart disease, stable angina and stroke. [12–14]. Most importantly, the earlier the effective interventions had been performed, the more significant the risk of cardiovascular disease and death would be reduced, with a reduction up to 15% [15].

Increased blood pressure is caused by a variety of factors, among which obesity is increasingly proved to be closely related to hypertension by accumulating evidence [16]. Previous studies mostly assessed obesity by using body mass index (BMI) and waist circumference (WC), however, obesity not only refers to excessive fat accumulation, but also implies the abnormal distribution of fat, and there are significant differences in morphology and function between subcutaneous adipose tissue (SAT) and visceral adipose tissue (VAT) [17]. Lipid accumulation product (LAP), as a combination of WC and triglycerides (TG), was proved to be an available indicator that reflects visceral obesity, and it was significant associated with total cholesterol, lipoproteins, and glucose [18, 19]. Higher LAP significantly increased the risk of hyperuricemia among Chinese rural population [20]. LAP was also proved to be significantly associated with insulin resistance, which involved in the development of a series of metabolic-related diseases [21]. Furthermore, LAP performed superior to BMI and WC as a predictor of metabolic syndrome [22]. However, to our best of knowledge, few articles applied LAP to assess obesity when analyzing the risk factors of prehypertension. Meanwhile, whether LAP performs better than other obesity indices for discriminating prehypertension has not been confirmed. Additionally, numerous studies have demonstrated that the interaction between environmental-genetic and environmental-environmental may be related to the occurrence of chronic diseases [23, 24]. For instance, the interaction between smoking and obesity has remarkable effects on type 2 diabetes risk in Chinese adults [23]. Therefore, the interaction between risk factors may aggravate the risk of prehypertension. However, most of previous studies only analyzed the risk factors for prehypertension, and rarely further explored their interactive effects.

This study, firstly, investigated the epidemiological characteristics of prehypertension and its associated factors. Secondly, the abilities of BMI, WC and LAP in predicting prehypertension risk were compared. Finally, we assessed the possibly interactive effects between various factors on prehypertension risk.

## Methods

### Study design

This community-based cross-sectional survey on the basis of the project called “creating a provincial demonstration area of chronic diseases management in community” was conducted in Longzihu, Bengbu, China. The project mainly aimed to investigate the epidemiological situation of main chronic diseases among residents living in Longzihu, Bengbu, China, and attempted to create a provincial demonstration community of chronic diseases management. Participants were selected through a multistage random sampling, which excluded individuals who had no abilities to communicate with investigators normally or finish the overall survey independently due to inconvenience or serious illness. After the screening, selected individuals were required to complete relevant survey and health checks in community clinics. All participants signed the informed consent. The Ethics Committee of Bengbu medical college approved this study.

### Questionnaire survey

A self-designed questionnaire as shown in Additional file 1 was completed for each participant by qualified staffs through face-to-face interview. The relevant definitions or grouping methods of sociodemographic variables were as followings: (1) Educational level: classified as “elementary school or lower”, “middle school graduate” and “high school graduate or higher”; (2) Monthly income: grouped as “0–2000”, “2000-” and “4000-” (yuan); (3) Marital status: categorized as “currently married” and “currently not married” (including single or divorced); (4) Smoking: defined by the status of pre-smoking or current-smoking; (5) Positive family history of cardiovascular diseases: refers to the individuals who had at least one parent or sibling with cardiovascular diseases [9]. Once the questionnaire survey was completed, the information was entered into Epidata software by using double entry approach.

### Blood pressure measurement

Before taking measuring blood pressure measurements, the participants were required to take a rest for 5 to 10 min. Afterwards, mercury sphygmomanometer was applied to measure blood pressure three times for each participant and the average one was calculated. Hypertension was defined as systolic blood pressure (SBP) ≥ 140 mmHg, or diastolic blood pressure (DBP) ≥ 90 mmHg, or the subject reported antihypertensive medication having been prescribed [25]. Individuals had SBP of 120–139 mmHg and/or DBP of 80–89 mmHg without antihypertensive medication were regarded as prehypertension [6], while those with SBP and DBP less than 120 mmHg and 80 mmHg respectively were defined as normotension [6].

### Anthropometric tests and laboratory examinations

Height, weight and WC were examined by trained investigators using uniform instruments. When measuring height and weight, the participants were required to take off their shoes and wear light clothes for obtaining more accurate measurements. BMI was calculated as weight(kg)/height(m)^2^. According to the recommendation given by the Working Group on Obesity in China, BMI ≥ 28 kg/m^2^ was defined as general obesity [26], WC ≥ 90 cm for males and WC ≥ 85 cm for females were regarded as abdominal obesity, respectively [27]. Meanwhile, all participants had blood samples taken after fasting for more than 8 h overnight. LAP was calculated as [WC (cm)-65] × [TG(mmol/L)] for males, and [WC (cm)-58] × [TG(mmol/L)] for females [18].

### Statistical methods

Firstly, the basic characteristics of enrolled participants were presented, and quantitative data and categorical variables were respectively described using mean ± SD (standard deviation) and percentages. Furthermore, the differences in categorical variables between normotension, prehypertension and hypertension individuals were compared by Chi-squared test or Kruskal-Wallis H test. LAP was divided into four groups (Q1, Q2, Q3, and Q4) in accordance with quartiles. Secondly, univariate and multivariate logistic regression model was applied to evaluate the risk factors for prehypertension, followed by the calculation of odds ratio (OR) with corresponding 95% confidence interval (95%CI). A stepwise backward selection procedure was used in multivariate analysis. Thirdly, the abilities of BMI, WC and LAP in predicting prehypertension risk were compared by the area under the receiver-operating characteristics (ROC) curves (AUC) analysis. Finally, the interaction between various factors on prehypertension was assessed by the following indicators: (1) the relative excess risk due to interaction (RERI = RR_11_- RR_10_-RR_01_ + 1); (2) the attributable proportion due to interaction (AP = RERI/RR_11_); (3) the synergy index (SI = (RR_11_–1)/ (RR_01_–1) + (RR_10_–1)) [28, 29]. All *p* values were two-sided, and *p* < 0.05 was considered statistically significant. R and Medcalc software were applied to complete all statistical calculations.

## Results

### Baseline characteristics

**Fig. 1:**
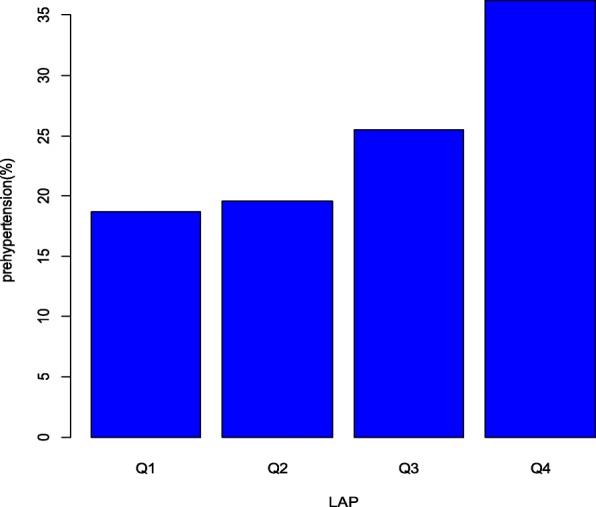
The prevalence of prehypertension across LAP quartiles (*P* for trend< 0.001)

Totally, 1777 middle-aged and elderly participants with average age of 60.82 were enrolled in this study, including 748 males (42.09%) and 1029 females (57.91%). The total prevalence of normtension, prehypertension and hypertension was 41.70%, 33.93% and 24.37% respectively, while male had a higher prevalence of prehypertension (37.43%) than female (31.39%). The mean age for normtension, prehypertension and hypertension members were 59.67 ± 11.34, 61.15 ± 11.41 and 62.31 ± 10.64 years old separately, with *p* < 0.01. Significant differences were presented in educational level (*p* = 0.03), smoking *(p* < 0.01), family history of cardiovascular diseases (*p* < 0.01), general obesity (*p* < 0.01) and abdominal obesity (*p* < 0.01) between normtension, prehypertension and hypertension individuals, among which the prehypertension members had the highest smoking rate (34.66%), intermediate prevalence of general obesity and abdominal obesity. However, no significant differences in marital status (*p* = 0.73) and income (*p* = 0.26) between groups were observed. As for LAP, a significant difference was obtained in LAP quartiles between three groups (*p* < 0.01), and the prevalence of prehypertension gradually increased *(p* < 0.01, trend Chi-square test) across LAP quartiles, as demonstrated in Fig. 1. All of the detailed information was presented in Table 1.

**Table 1:** Basic characteristic of participants in this study

Variables	Normotension (*N* = 741)	Prehypertension (*N* = 603)	Hypertension(*N* = 433)	*P* ^*a*^
Gender (%)				< 0.01^b^
Male	255(34.41)	280(46.43)	213(49.19)	
Female	486(65.59)	323(53.57)	220(50.81)	
Age (years)	59.67 ± 11.34	61.15 ± 11.41	62.31 ± 10.64	< 0.01^c^
Educational level (%)				0.03^c^
Elementary level or lower	233(31.44)	189(31.34)	168(38.80)	
Middle school graduate	276(37.25)	221(36.65)	146(33.72)	
High school graduate or higher	232(31.31)	193(32.01)	119(27.48)	
Marital status (%)				0.73^b^
Currently married	621(83.81)	506(83.91)	370(85.45)	
Currently not married	120(16.19)	97(16.09)	63(14.55)	
Income (yuan) (%)				0.26^c^
0–2000	419(56.55)	315(52.24)	230(53.12)	
2000–4000	288(38.87)	256(42.45)	185(42.73)	
> 4000	34(4.58)	32(5.31)	18(4.15)	
Smoking (%)				< 0.01^b^
No	573(77.33)	394(65.34)	284(65.59)	
Yes	168(22.67)	209(34.66)	149(34.41)	
Family history of cardiovascular diseases (%)				< 0.01^b^
No	608(82.1)	458(75.95)	322(74.36)	
Yes	133(17.9)	145(24.05)	111(25.64)	
General obesity (%)				< 0.01^b^
No	660(89.05)	505(83.75)	324(74.83)	
Yes	81(10.95)	98(16.25)	109(25.17)	
Abdominal obesity (%)				< 0.01^b^
No	502(67.75)	304(50.41)	151(34.87)	
Yes	238(32.25)	299(49.59)	282(65.13)	
LAP (%)				< 0.01^c^
Q1	226(30.50)	113(18.74)	42(9.70)	
Q2	215(29.01)	118(19.57)	92(21.25)	
Q3	182(24.56)	154(25.54)	119(27.48)	
Q4	118(15.93)	218(36.15)	180(41.57)	

a:Comparisons of variables between normotension, prehypertension and hypertension members

b:Chi-squared test

c: Kruskal-Wallis H test

### Analyses of risk factors for prehypertension

The results of univariate and multivariate logistic regression analysis were introduced in Table 2. Male had a higher risk of prehypertension than female in univariate analysis (OR: 1.65, 95%CI: 1.32–2.06), but no association of importance was observed after controlling other factors (OR: 1.16, 95%CI: 0.85–1.58). Besides, both univariate and multivariate analysis indicated that individuals had a higher risk of being prehypertension with aging (OR: 1.01, 95%CI: 1.00–1.02). No statistically significant relationship between educational level, income and marital status with prehypertension risk were observed. Compared with non-smoker, smokers had 1.67 fold risks in getting prehypertension, testified by multivariate analysis (OR: 1.67, 95%CI: 1.22–2.29). Members with positive family history of cardiovascular diseases were similarly effected in prehypertension (OR: 1.52, 95%CI: 1.14–2.02). In terms of obesity indices, a significant association between general obesity and increased risk of prehypertension were detected using both univariate (OR: 1.58, 95%CI: 1.15–2.17) and multivariate analysis (OR: 1.51, 95%CI: 1.15–1.97). However, in univariate analysis, abdominal obesity was significantly related to prehypertension (OR: 2.08, 95%CI: 1.67–2.60), while in multivariate analysis, there was no correlation worth of attention (OR: 1.94, 95%CI: 0.89–1.60).The risk of prehypertension significantly increased with LAP levels in the fourth quartile as compared with the bottom quartile (crude OR: 3.70, 95%CI: 2.69–5.08; adjusted OR: 2.58, 95% CI: 1.76–3.80).

**Table 2:** Logistic regression model for risk factors associated with prehypertension

Variables	Univariate analysis		Multivariate analysis	
	OR	95%CI	OR	95%CI
Gender				
Female	1.00(ref.)	–	1.00(ref.)	–
Male	1.65	1.32–2.06	1.16	0.85–1.58
Age (years)	1.01	1.00–1.02	1.01	1.00–1.02
Educational level				
Elementary level or lower	1.00(ref.)	–	1.00(ref.)	–
Middle school graduate	0.99	0.76–1.28	0.99	0.74–1.33
High school graduate or higher	1.03	0.78–1.34	1.24	0.90–1.69
Marital status				
Currently married	1.00(ref.)	–		
Currently not married	0.99	0.74–1.33	0.99	0.72–1.37
Income (yuan)				
0–2000	1.00(ref.)	–	1.00(ref.)	–
2000–4000	1.18	0.95–1.48	0.98	0.76–1.27
> 4000	1.25	0.76–2.07	0.87	0.49–1.53
Smoking	1.81	1.42–2.30	1.67	1.22–2.29
Family history of cardiovascular diseases	1.45	1.11–1.89	1.52	1.14–2.02
General obesity	1.58	1.15–2.17	1.51	1.15–1.97
Abdominal obesity	2.08	1.67–2.60	1.94	0.89–1.60
LAP				
Q1	1.00(ref.)	–	1.00(ref.)	–
Q2	1.10	0.88–1.51	0.96	0.68–1.34
Q3	1.69	1.24–2.31	1.32	0.93–1.87
Q4	3.70	2.69–5.08	2.58	1.76–3.80

### Comparisons between LAP, BMI and WC

**Fig. 2:**
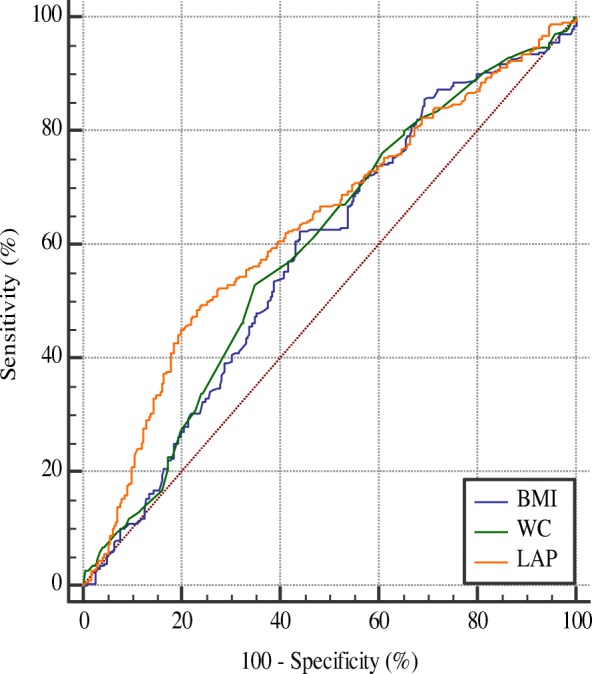
The ROC curve of obesity indices in predicting prehypertension risk in males

**Fig. 3:**
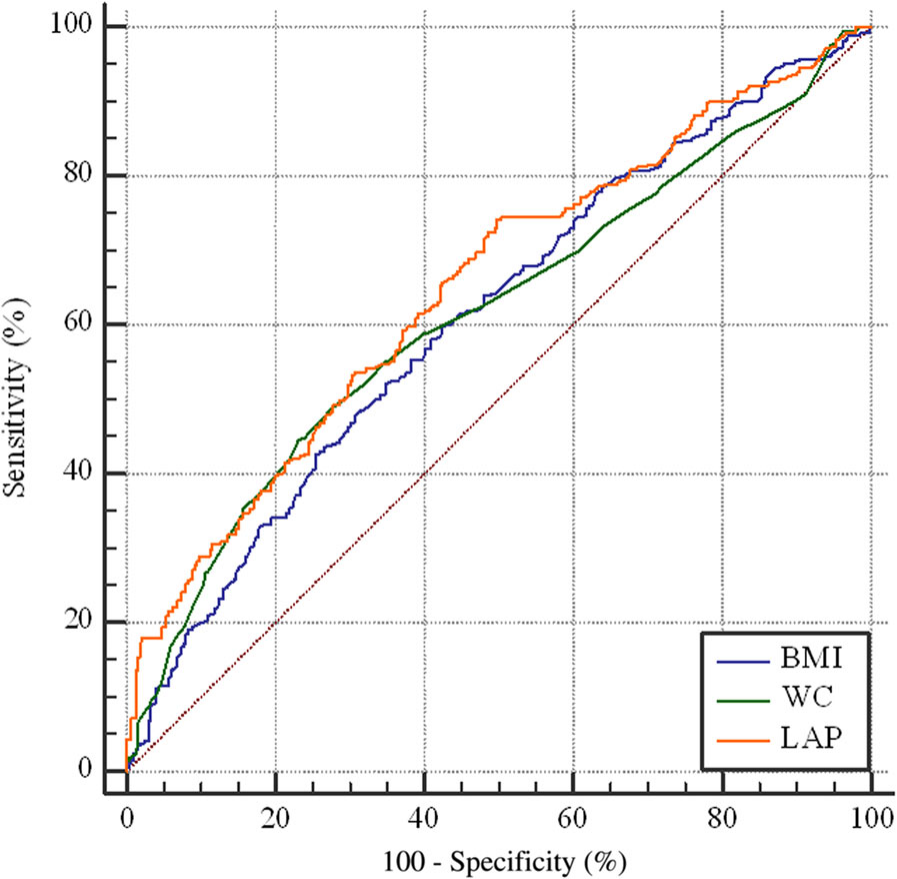
The ROC curve of obesity indices in predicting prehypertension risk in females

The ROC curves analyses were presented in Table 3 and Figs. 2 and 3. Overall, the AUC with corresponding 95%CI of BMI, WC and LAP were 0.60(0.57–0.63), 0.63(0.60–0.65) and 0.65 (0.62–0.68) respectively. LAP performed better than BMI (Z = 3.52, *P* < 0.01) and WC (Z = 2.05, *P* = 0.04) in discriminating prehypertension risk. Moreover, when grouped by gender, although LAP still performed better than BMI in male (Z = 2.05, *P* = 0.03) and female (Z = 2.12, *P* = 0.03), its AUC was significant higher than that of WC only in female (Z = 2.43, *P* = 0.01), but not in men (Z = 1.77, *P* = 0.07). The AUC with corresponding 95%CI of BMI, WC, LAP were 0.59(0.54–0.63), 0.60(0.55–0.64), 0.63(0.59–0.67) in males, and 0.61(0.58–0.65), 0.61(0.58–0.65), 0.65(0.62–0.68) in females respectively.

**Table 3:** the comparisons of obesity indices in predicting prehypertension risk

		Cut-off value	Sensitivity (%) *P*^a^	Specificity (%)	AUC(95%CI)	*Z*	
All	BMI	23.99	61.03	57.09	0.60(0.57–0.63)	3.52	< 0.01
	WC	86.50	52.40	68.83	0.63(0.60–0.65)	2.05	0.04
	LAP	38.22	56.22	66.80	0.65(0.62–0.68)	–	–
Male	BMI	24.00	62.50	56.08	0.59(0.54–0.63)	2.05	0.03
	WC	88.00	53.21	65.10	0.60(0.55–0.64)	1.77	0.07
	LAP	48.18	49.29	76.47	0.63 (0.59–0.67)	–	–
Female	BMI	23.95	59.75	57.61	0.61(0.58–0.65)	2.12	0.03
	WC	86.50	44.58	76.95	0.61(0.58–0.65)	2.43	0.01
	LAP	26.40	74.30	50.21	0.65 (0.62–0.68)	–	–

^a^compared with AUC

### Interaction analysis

Finally, the interaction analyses were conducted by relevant indicators as shown in Table 4, and the interaction between LAP and family history of cardiovascular diseases on prehypertension risk was found to be significant (RERI: 1.88, 95%CI: 0.25–3.51; AP: 0.44, 95%CI: 0.20–0.69; SI: 2.37, 95%CI: 1.22–4.60). According to the results of RERI (1.99, 95%CI: 0.04–3.93), AP (0.42, 95%CI: 0.17–0.67) and SI (2.16, 95%CI: 1.68–4.00), LAP also considerably associated with smoking in prehypertension risk. The value of AP (0.40, 95%CI: 0.03–0.77) indicated a significant interaction of family history of cardiovascular diseases and smoking on prehypertension, while neither RERI (1.32, 95%CI:-0.60–3.23) nor SI did (2.37, 95% CI: 0.87–6.44).

**Table 4:** the interaction analysis for prehypertension risk

Variable		Interaction analysis^b^		
		RERI	AP	SI
LAP^a^	Family history	1.88(0.25–3.51)^3^	0.44(0.20–0.69)^3^	2.37(1.22–4.60)^3^
LAP^a^	Smoking	1.99(0.04-3.93)^3^	0.42(0.17-0.67)^3^	2.16(1.68–4.00)^3^
Family history	Smoking	1.32(−0.60–3.23)^4^	0.40(0.03-0.77)^3^	2.37(0.87-6.44)^4^

^a^: grouped by cut-off values in Table 2

^b^: adjust for age, sex, educational level, marital status and monthly income

^3^: *P*<0.05

^4^: *P*>0.05

## Discussion

With the rapid economic and social development, the number of Chinese adults with prehypertension has become considerably massive [30–34]. The prevalence of prehypertension (33.93%) in this study was consistent with the survey conducted in Taiwanese adults (34.0%) [30], higher than that of adults living in Inner Mongolia (28.77%) [10], Jiangxi province (32.3%) [31] and Zhejiang province (32.1%) [32], but lower than the rate in Hubei Province (42.2%) [33] and Qinghai Province (41.3%) [34]. China is a vast multi-ethnic country, consequently, the specific life styles and distinct socioeconomic status may influence the epidemic of prehypertension. In other Asian countries, the National Adult Overweight Survey 2005 in Vietnam reported a prehypertension prevalence rate of 41.8% in 17,199 adults [35]. Moreover, Lifestyle Promotion Project (LPP) in Iranian population announced that the prevalence of prehypertension was as high as 47.3% [36], while Korean National Health and Nutrition Examination Survey (KNHANES) demonstrated a similar rate of prehypertension (33.3%) with our research [37]. In the United States, a cohort study named Reasons for Geographic and Racial Differences in Stroke (REGARDS) reported an amazing prevalence of prehypertension of 62.9% in black and 54.1% in white participants [38]. Numerous researches have demonstrated that prehypertension has serious effects on human health such as carotid atherosclerotic plaque [39], stroke [14] and even mortality [40]. Thus, prehypertension is a prevalent public health problem that worth attention worldwide. The present study also revealed that the prevalence of prehypertension significantly increased with aging, suggesting that the prevention of prehypertension should be carried out as early as possible. Similarly, Liu et al. [41] conducted a survey with 3891 Chinese adults, and the results also demonstrated that age was a significant risk factor of prehypertension in both genders. In contrast with the univariate analysis, the risk of prehypertension in male was not significantly higher than female, proposing that the influence of gender on prehypertension remains inconsistent, with several studies reporting a significant relationship [6, 38], while others did not [31]. This may be resulted by the differences in ethnic group and various adjusted variables in different studies.

The commonness of obesity in China has increased dramatically in recent years [42]. Extensive studies have proved that obesity, especially visceral obesity, plays an essential role in the increase of blood pressure [16]. Visceral fat can activate the renin-angiotensin-aldosterone system [43]. The aldosterone concentration is positively correlated with the amount of visceral adipose tissue as VAT can stimulate the release of aldosterone from adrenal cells [44]. Insulin metabolism may be affected by visceral fat through releasing free fatty acids. Meanwhile, visceral fat is capable to promote inflammations process through a source of adipokines, such as tumor necrosis factor-alpha (TNF-alpha), plasminogen activator inhibitor-1 and angiotensinogen and C-reactive protein [45].

Traditional obesity indices, including BMI and WC, have certain limitations. For instance, BMI is unable to distinguish between fat and muscle, and is not suitable to evaluate the people whose muscle accounts for a larger proportion of the body composition. Recently, “Obesity paradox” has attained notable attention. Heart failure, chronic kidney disease, or cancer patients with obesity defined by BMI have even a better prognosis than those with normal weight [46–48]. WC can accurately reflect abdominal obesity, but is unable to distinguish between subcutaneous fat and visceral fat. Importantly, visceral and subcutaneous adipose depots play differential roles in human health [17, 49]. Substantial evidence have suggested that visceral obesity may be more closely related with adverse outcomes such as cardiovascular diseases and death, and higher VAT significantly reduced the probability of conversion of prehypertension transforming to normotension [49–52]. Compared with SAT, VAT adipocytes are more metabolically active and less sensitive to insulin than SAT, it also can generate more free fatty acids and has a greater ability to uptake glucose [53]. The Framingham Heart study with a follows up lasted for 6.2 years demonstrated that the effect of SAT on metabolic risk factors was less striking than that of VAT [50]. Tang et al. [51] measured VAT and SAT among 1449 Chinese adults by MRI, and the results also indicated that VAT was more strongly associated with cardiometabolic risk factors than SAT. High visceral fat with low subcutaneous fat accumulation was significantly related with atherosclerosis in type 2 diabetes patients, suggesting that SAT may be a protective role against atherosclerosis [54]. Compared with epicardial fat volume and SAT, VAT had the strongest effect on cardiometabolic diseases [55]. Madero et al. [56] compared the value of different measures of body fat, including SAT, VAT, BMI and WC in predicting the incidence of chronic kidney diseases, and only VAT remained a decisive factor in multivariable analysis. Unfortunately, neither of BMI nor WC can distinguish between subcutaneous fat and visceral fat. Meanwhile, computed tomography (CT) and magnetic resonance imaging (MRI), as the gold standards to evaluate visceral fat, are not appropriate for widespread promotion and use in large-scale epidemiological survey because of their high costs and radiation exposure. Consequently, an inexpensive, efficient and available indicator that reflects visceral obesity is urgently needed.

LAP, as a combination of WC and TG, can reflect anatomic and physiologic changes and has theoretical basis to evaluate visceral obesity [18]. TG can reflect the degree of visceral fat accumulation caused by metabolic disorders, and significant relationships had been found between WC and insulin resistance, hypertension and metabolic syndrome [57–59]. Moreover, a cross-sectional study in China demonstrated that higher TG was the main risk factor of prehypertension [41]. Surprisingly, no significant relationship was found between WC and prehypertension risk in multivariable model, which was consistent with a published study [31]. This may be explained by the fact that most of individuals with abdominal obesity would progress to actual hypertension [31]. A strong correlation between LAP and area of visceral adipose tissue measured by CT were observed, suggesting that LAP was an effective marker discriminating visceral obesity [60]. “Hypertriglyceridemic waist (HTGW)”, the combination of WC and TG, is a dichotomous indicator, while LAP is developed to express as a continuous indicator. A growing number of evidence has proved that LAP may be more reasonable and scientific than HTGW, as obesity itself is a continuous process [18]. As a result, LAP was applied as a visceral obesity indicator rather than HTGW in this study.

The results obtained in this study indicated that higher LAP significantly increased the risk of prehypertension, and it is superior to BMI and WC for discriminating prehypertension risk. However, when grouped by gender, LAP was only better than WC in female. With aging, the patterns of lipid over accumulation in men and women became more and more distinct, as LAP got higher or stayed the same level in women with aging, while it reduced gradually in men at older age [61]. At the same BMI level, female have more body fat than male [62]. In addition to the greater impact of Hyper-TG on cardiovascular diseases in female than male [63], VAT was reported to be more strongly associated with cardiometabolic risk factors in obese female than male as well [64]. In men, SAT and VAT had similar effects on insulin resistance, while in female, only VAT was associated with insulin resistance [65]. Similar to our study, LAP seemed to increase diabetes risk stronger in female than male in Japanese [66]. What is more, a hospital-based cross-sectional survey in China indicated that LAP was significantly associated with intracranial atherosclerotic stenosis in middle-aged and elderly female, but not in male [67]. Compared with BMI, LAP performed better in identifying chronic kidney disease in female living in the rural of Northeast China, but less well in male [68]. Therefore, LAP may have a more excellent value in female and the gender-specific differences need to be further explored. Additionally, several studies have also demonstrated that the relationship between LAP and diseases risk may be influenced by age, which was then proved by the analysis of the relationship between LAP and risk of non-alcoholic fatty liver disease in Chinese adults, indicating that the diagnostic ability of LAP was higher in younger adults [69]. A cohort study with 6-years follow-up further explored that LAP was superior to BMI in predicting incident diabetes only in young men [70]. A given WC represented differential amount of visceral fat in older subjects and younger subjects [71]. However, in this study, the mean age of participants was 60.82, for that reason, the effect of LAP on prehypertension in younger groups should be further investigated.

Biological interaction refers to the mutual influence of two pathogenic factors on the pathogenesis of the diseases. We demonstrated significant interactions between LAP and family of cardiovascular diseases, LAP and smoking, family of cardiovascular diseases and smoking on prehypertension risk, separately. Family history of cardiovascular diseases is considered to be an indicative sign of genetic susceptibility. The present study, as well as other research [9], announced a significant relationship between family of cardiovascular diseases and prehypertension risk. Laboratory stressor tests showed that healthy subjects with family history of cardiovascular diseases had a more positive hemodynamic responsiveness to stressor tests [72]. Our results demonstrated that smokers had 1.67 fold risks in getting prehypertension, while other researchers failed to report a significant relationship [31, 34]. This may be due to the various standards of smoking. It was suggested by JNC-7 that lifestyle modifications, including quitting smoking, might be beneficial to the prevention of prehypertension. Furthermore, relevant research had indicated that obesity and smoking have several common mechanisms to increase blood pressure, such as inhibiting vascular reflex vasodilation and increasing oxidative stress [73]. Smoking was interacted with obesity on diabetes risk [23]. There was also a significant interaction of passive smoking with pregnancy obesity on risk of gestational diabetes mellitus in Chinese adults [74]. So far, there are few articles exploring the interaction of risk factors on prehypertension risk, and the interactive mechanisms between factors needs to be further studied in the future.

There are several limitations of this study in the following aspects. Firstly, as a cross-sectional survey, it failed to infer causality in its results. Secondly, there were ethnic and racial differences in body composition [75], therefore the relationship between LAP and prehypertension was not clear in other ethnic individuals. Thirdly, whether participated individuals had taken lipid lowering drugs was not investigated.

## Conclusion

In conclusion, prehypertension is prevalent in Chinese adults. This study indicated that age, family history of cardiovascular diseases, smoking and LAP were significantly related to prehypertension risk. Furthermore, we demonstrated significant interactions between risk factors on prehypertension risk, which would help us to establish strategy against prehypertension more comprehensively and scientifically. Further studies should pay more attention to the gender-difference of LAP and the underlying mechanisms of interactive effect on prehypertension risk.

## Additional file

Additional file 1: Survey questionnaire in English. (DOCX 17.9 kb)

## Abbreviations

BMI: Body mass index
CT: Computed tomography
LAP: Lipid accumulation product
MRI: Magnetic resonance imaging
SAT: Subcutaneous adipose tissue
VAT: Visceral adipose tissue
WC: Waist circumference
