# Supplementary material for: Risk factors for prehypertension and their interactive effect: a cross- sectional survey in China
Source: BMC Cardiovasc Disord. 2018 Sep 15;18:182. doi: 10.1186/s12872-018-0917-y (PMC6139180; doi:10.1186/s12872-018-0917-y)
Supplement: Supplementary file 1 — Survey questionnaire in English. (DOCX 17.9 kb) [file 12872_2018_917_MOESM1_ESM.docx]

**Questionnaire**

**A1.** Name：

Home address：

**A2.** Your age： years old（birth data： year month, based on the information on the ID card）

**A3.** Gender： 1. Male 2.Female

**A4.** Your educational level?

1. Elementary school or lower 2. Middle school graduate 3. High school graduate or higher (including special secondary school and technical school)

**A5.** Your current marital status?

1. Currently not married (including divorce and widowhood)

2. Currently not married

**A6.** Your family income monthly？

1. 0-2000 2. 2000-4000 3. 4000-

**A7.** Do you have a family history of cardiovascular diseases？

1. Yes 2. No

**A8.** Do you smoke？________

1. Current-smoking 2. Pre-smoking 3. Never-smoking
